# Dysbiosis not observed in Canadian horses with free fecal liquid (FFL) using 16S rRNA sequencing

**DOI:** 10.1038/s41598-024-63868-1

**Published:** 2024-06-05

**Authors:** Robert J. Wester, Lyndsey L. Baillie, Garrett C. McCarthy, Carson C. Keever, Lauren E. Jeffery, Paul J. Adams

**Affiliations:** 1 Applied Genomics Centre, Kwantlen Polytechnic University, Surrey, BC Canada; 2 Faculty of Science, Kwantlen Polytechnic University, Surrey, BC Canada; 3 Agwest Veterinary Group Ltd., Abbotsford, BC Canada

**Keywords:** Animal physiology, Bacterial genetics, Classification and taxonomy, Dysbiosis

## Abstract

Free Fecal Liquid (FFL), also termed Fecal Water Syndrome (FWS), is an ailment in horses characterized by variable solid and liquid (water) phases at defecation. The liquid phase can be excreted before, during, or after the solid defecation phase. While the underlying causes of FFL are unknown, hindgut dysbiosis is suggested to be associated with FFL. Three European studies investigated dysbiosis in horses with FFL using 16S rRNA sequencing and reported results that conflicted between each other. In the present study, we also used 16S rRNA sequencing to study the fecal microbial composition in 14 Canadian horses with FFL, and 11 healthy stable mate controls. We found no significant difference in fecal microbial composition between FFL and healthy horses, which further supports that dysbiosis is not associated with FFL.

## Introduction

Free Fecal Liquid (FFL), also known as fecal water syndrome (FWS), is a condition in domestic horses in which defecation occurs in both a liquid and solid phase^1–3^. The liquid phase can occur either before, during, or after defecation^4^. Horses with FFL sometimes display signs of discomfort during defecation^2^ and persistently have soiled hindquarters^1^, as well as a high risk of developing dermatitis, bloated abdomen, and weight loss in severe cases^2,3^. While the exact causes of FFL are unknown, several factors including social stress^1,2^ endoparasitic infection, poor dentition, and diet have been suggested as at least being contributing factors^5^. Diet^6^, environmental conditions^6,7^ and stress^4,6,8^ also have a significant impact on the composition of the microbial population (microbiota) within the intestines. An imbalance in equine gut microbiota composition (dysbiosis) is already well documented to affect the normal health and function of the gastrointestinal tract and is associated with diseases like colic^9^, colitis^10^, laminitis^11,12^, and diarrhea in foals^13,14^. As such, dysbiosis has been suspected to play a significant role in FFL.

Recent studies have compared the microbial composition of horses with FFL to healthy control subjects and found conflicting results. Two studies in particular, Lausten et al.^1^ and Schoster et al.^3^, concluded no statistical difference in microbial composition between healthy horses and those with FFL. However, another study found statistically significant differences in the population contribution of genera *Alloprevotella* and *Bacillus* between FFL horses and stable-matched controls, while the overall population diversity of the microbiota remained similar between the groups^5^. Notably, all previous studies regarding the relationship between FFL and the microbiome have been carried out in Europe. Therefore, the objective of this study was to expand the current understanding of FFL and extend sampling to North America, by examining fecal microbiome profiles of both healthy horses and those exhibiting FFL in the Fraser Valley region of British Columbia, Canada. Given the reported associations of dysbiosis with other gastrointestinal tract diseases, we hypothesized that dysbiosis may also be associated with FFL in Canadian horses. However, our results support the conclusion that there is no significant difference between the microbiome of feces from FFL animals and controls.

## Methods

### Animals and sample collection

A case-controlled study was performed by sampling horses suffering with FFL and healthy stable matched controls. Fecal samples were collected and provided by an Agwest Veterinary Group Ltd. veterinarian in Fall 2020 during routine care of horses distributed across British Columbia’s Fraser Valley. The veterinarian collected approximately 40 mL of feces from each horse using the rectal grab technique. During collection, the health status, age, breed, sex, feed, and farm location were also noted. Following collection, the fecal samples were immediately placed on ice and kept for a maximum of eight hours before storing at -80°C until DNA extraction.

### DNA Extraction and 16S rRNA sequencing

DNA was extracted from fecal samples using the QIAamp PowerFecal DNA Kit (Qiagen) according to the manufacturer’s instructions. DNA quality was assessed by NanoDrop™ One Spectrophotometer (Thermo Scientific™) and DNA quantity was determined using the Qubit 4 Fluorometer (Invitrogen).

DNA was amplified using PCR targeting multiple hypervariable regions of the 16S rRNA bacterial gene using the V2-4–8 and V3-6,7–9 primer sets included with the Ion 16S™ Metagenomics Kit (Life Technologies). Amplicons were purified by Agencourt® AMPure® XP Kit (Beckman Coulter) and subsequently pooled by sample. Library preparation was conducted using the Ion Plus Fragment Library Kit (Thermo Fisher Scientific) according to the manufacturer’s recommendations for barcoded libraries using the Ion Xpress™ Barcode Adapters 1–96 Kit (Thermo Fisher Scientific). Libraries were quantified using the Ion Universal Library Quantification Kit (Life Technologies), normalized to 10pM, and then pooled. Template generation occurred within the Ion Chef instrument (Ion Torrent™) using the pooled libraries and an Ion 520™ Chip (Ion Torrent™). The loaded Ion 520™ Chip was sequenced using the Ion GeneStudio™ S5 System (Ion Torrent™). All extraction, library preparation, and sequencing steps were performed at the Applied Genomics Centre at Kwantlen Polytechnic University in Surrey, BC.

### Statistical analysis

Sequence data was analysed using the QIIME2 pipeline, as outlined in the QIIME2 documentation (https://docs.qiime2.org/2023.5/)^16^. Demultiplexed fastq files collected from the GenoStudio™ S5 System were imported into QIIME2 using the Single End Fastq Manifest Phred 33V2 format. Sequences were filtered by quality scores and ambiguous base calls^17^, then dereplicated using VSEARCH^18^ to produce a feature table and feature representative sequences. Contigs were aligned to SILVA 16S rRNA (138 release) sequence reference database^19,20^ and classified as operational taxonomic units (OTUs) using the closed reference feature clustering approach^18^. Chimeric representative sequences were detected using UCHIME^18^ and filtered out. The OTUs were classified against the SILVA 16S rRNA (138 release) sequence reference database^19,20^ using the VSEARCH-based consensus taxonomy classifier pipeline^18^ within the QIIME2 feature-classifier plugin^21^. Representative sequences were used to construct a rooted phylogenetic tree using the MAFFT-FastTree pipeline^22,23^. The OTU feature table, taxonomic feature data, and rooted phylogenetic tree QIIME2 artifacts were exported.

The exported OTU feature table, taxonomic feature data, rooted phylogenetic tree, and the sample metadata file were imported into R^24,25^ loaded with the phyloseq^26^, vegan^27^, and tidyverse^28^ packages. The files were used as components to construct a phyloseq object, which was subsequently filtered based on taxonomic classification status^26^. The phyloseq object was used to display the relative abundance of each taxonomic classification with a mean above 1% for comparison^28^. Normality of the data was tested using the Shapiro-Wilks test^29^. Abundances were compared between FFL status, sex, and geographical location using the Kruskal–Wallis test^30^ and the Wilcoxon rank sum test^31^, using the Benjamini–Hochberg procedure using α = 0.05^32^ to adjust for false discovery rates (FDR). The phyloseq object was then rarefied for downstream diversity metrics testing^26^. Alpha diversities were quantified using the Shannon index^26,33^, Simpson index^26,34^, and Chao richness^26,35^ and were compared by FFL status, sex, and geographical location using the Kruskal–Wallis test^30^ and Wilcoxon rank sum hypothesis testing^31^. The Benjamini–Hochberg procedure using α = 0.05^32^ of adjustment was used to control FDR. Beta diversity, the dissimilarity in community composition, was quantified for FFL status and geographical locations using the Bray–Curtis index^27,36^ and Jaccard distance^27^. Quantified beta diversity metrics were compared using one-way permutational multivariate analysis of variance (PERMANOVA)^37^ and analysis of similarities (ANOSIM)^38^, each using 999 permutations. The dissimilarity between FFL status, sex, and geographical location was visualized by principal coordinate analysis (PCoA) plots^27,28^.

#### Ethics declaration

The samples were collected by an Agwest Veterinary Group Ltd. veterinarian in accordance with the Canadian Association for Laboratory Animal Medicine (CALAM) Standards of Veterinary Care, which adheres to the ARRIVE guidelines^15^.

## Results

### Animal demographic data

A total of 25 horses were examined, 14 horses with FFL and 11 stable-mate controls. Among the affected horses, five were male. Among the control group, seven were male. The horses sampled were from one of six geographical locations within British Columbia’s Fraser Valley: Richmond (7), Surrey (2), Langley (6), Maple Ridge (1), Abbotsford (7), and Matsqui Flats (2). The horses included in the study had a mean age of 14 and a half years and a range of 2 to 29 years. Detailed information on horse metadata is presented in Supplementary Table S1.

### Sequencing output

Sequencing analysis of the 16S rRNA gene yielded a total of 208,907 sequence reads that passed all quality control filters. All 25 samples returned more than 1,000 reads and were retained for further analyses and had a median sequencing depth of 7,963 reads. Specifically, the control horses had a total of 97,754 sequencing reads (median; 8,114 reads), while the FFL horses had 111,153 sequencing reads (median: 7,837 reads).

### Microbial population composition

**Figure 1:**
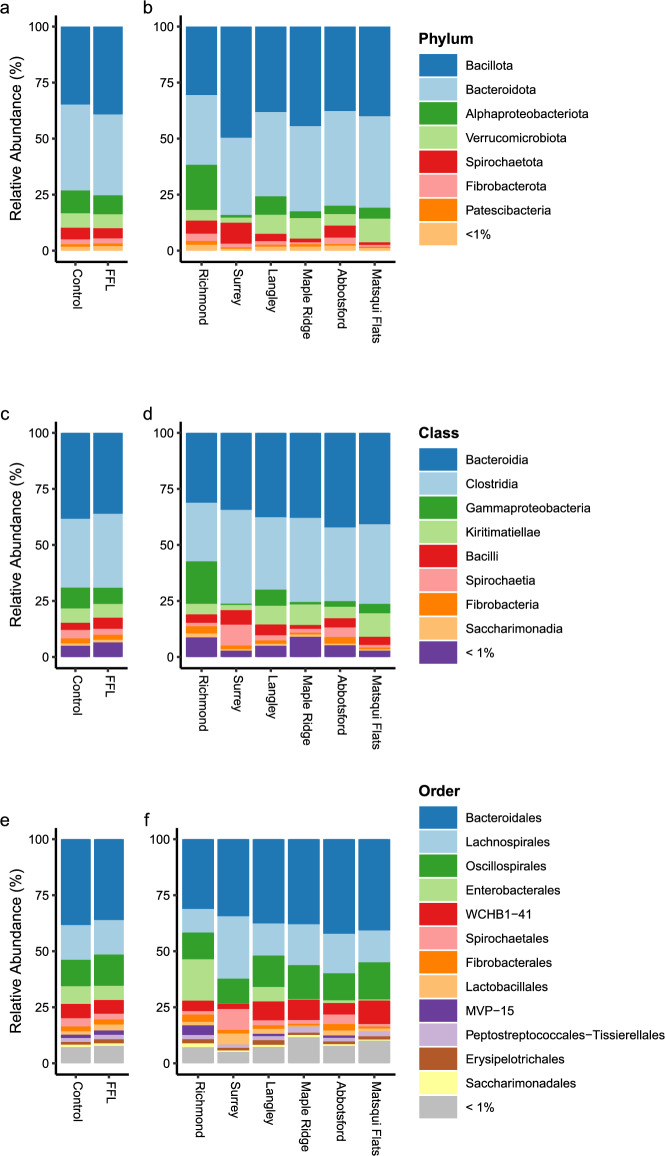
Bar charts illustrating the mean relative abundance of phyla (**a** and **b**), classes (**c** and **d**), and orders (**e** and **f**) between FFL status (**a**, **c**, and **e**) and geographical location (**b**, **d**, and **f**). All named taxa displayed have a mean relative abundance above 1%.

The microbial population for all 25 horses in the study was determined. We identified 7 phyla, 8 classes, 12 orders, 17 families, and 17 genera which exhibited a relative abundance greater than 1%. The seven most abundant phyla are Bacillota (median: 37.155 ± 12.465%), Bacteroidota (median: 37.838 ± 10.429%), Alphaproteobacteriota (median: 3.986 ± 6.874%), Verrucomicrobiota (median: 5.657 ± 5.025%), Spirochaetota (median: 3.968 ± 4.316%), Fibrobacterota (median: 1.442 ± 1.857%), and Patescibacteria (median: 0.940 ± 0.691%). The most abundant phyla, classes, and orders between FFL status and geographic location are presented in Fig. 1. The most abundant families and genera are displayed in Supplementary Fig. 1. Relative abundances above 1% at all classification levels for each horse in the study are presented in Supplementary Fig. 2. Additionally, the most abundant taxa at all classification levels between sex are depicted in Supplementary Fig. 3.

### Alpha diversity

**Figure 2:**
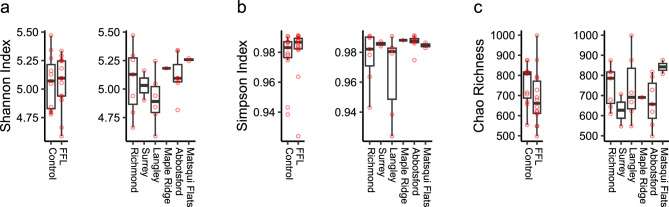
Boxplots overlayed with all data points of alpha diversity index measure estimates using Shannon Evenness (**a**), Simpson Diversity (**b**), and Chao Richness (**c**) between FFL status and geographical locations groupings. Alpha diversity indices between groups did not show statistical significance (*p* > 0.05).

Alpha diversity indices between horses with FFL and the control group were compared using the Wilcoxon rank sum test and found no statistically significant differences in Shannon index (*p* = 0.767), Simpson index (*p* = 0.999), and Chao richness (*p* = 0.809). Furthermore, the alpha diversity indices between geographical location were compared using the Kruskal–Wallis test and found no statistically significant differences in Shannon index (*p* = 0.571), Simpson index (*p* = 0.059), and Chao richness (*p* = 0.201). Boxplots of all diversity indices for both FFL status and geographical location are outlined in Fig. 2.

### Beta diversity

**Figure 3:**
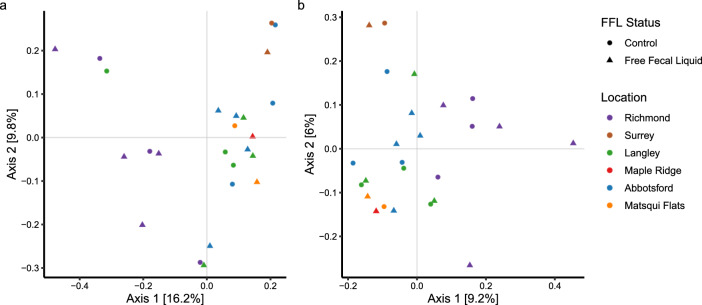
Principal coordinate analyses (PCoA) of the microbial composition and diversity between FFL status and geographical locations using the Bray–Curtis index (**a**) and the Jaccard index (**b**). Circular icons indicate healthy control horses and triangular icons indicate those with FFL. Each colour represents a different geographical location.

Based on the Bray–Curtis and Jaccard PCoA plots (Fig. 3), no distinct clusters were visually evident between FFL horses and healthy controls. This was confirmed through ANOSIM and PERMANOVA analyses, which indicate no significant difference in community composition and structure (Table 1, Bray–Curtis ANOSIM: *p* = 0.829, Jaccard ANOSIM: *p* = 0.638, Bray–Curtis PERMANOVA: *p* = 0.904, Jaccard PERMANOVA: *p* = 0.934). Additionally, we compared overall differences between sex and also found no statistical differences between groups (Table 1, Bray–Curtis ANOSIM: *p* = 0.556, Jaccard ANOSIM: *p* = 0.238, Bray–Curtis PERMANOVA: *p* = 0.411, Jaccard PERMANOVA: *p* = 0.207). The PCoA plots outlining the differences between sex using the Bray–Curtis and Jaccard indices is depicted in Supplementary Fig. 4. However, significant difference was observed when comparing overall geographical location (Table 1, Bray–Curtis ANOSIM: *p* = 0.002, Jaccard ANOSIM: *p* = 0.001, Bray–Curtis PERMANOVA: *p* = 0.001, Jaccard PERMANOVA: *p* = 0.001). Furthermore, relative differences between the Richmond and Abbotsford sample communities are evident in the PCoA plots (Fig. 3). This was confirmed statistically by Bray–Curtis pairwise ANOSIM (*p* = 0.015, R = 0.43), Jaccard pairwise ANOSIM (*p* = 0.015, R = 0.39), Bray–Curtis pairwise PERMANOVA (*p* = 0.015, R^2^ = 0.167, F = 2.422) and Jaccard pairwise PERMANOVA (*p* = 0.015, R^2^ = 0.103, F = 1.376) analysis. Other geographical location combinations did not indicate pairwise statistical significance for either ANOSIM or PERMANOVA analyses (*p* > 0.05).

**Table 1:** Overall differences in bacterial microbial composition and structure using the Bray–Curtis and Jaccard diversity indices between FFL status, sex, and geographical location using ANOSIM and PERMANOVA.

		ANOSIM		PERMANOVA		
		*P*-value	R	*P*-value	R^2^	F
FFL Status	Bray–Curtis	0.829	− 0.055	0.904	0.032	0.772
	Jaccard	0.638	− 0.024	0.934	0.038	0.905
Sex	Bray–Curtis	0.556	− 0.015	0.411	0.042	1.007
	Jaccard	0.238	0.036	0.207	0.044	1.054
Geographical location	Bray–Curtis	0.002	0.311	0.001	0.279	1.468
	Jaccard	0.001	0.311	0.001	0.251	1.273

ANOSIM, analysis of similarities; PERMANOVA, permutational multivariate analysis of variance.

### FFL Status microbial composition comparison

**Figure 4:**
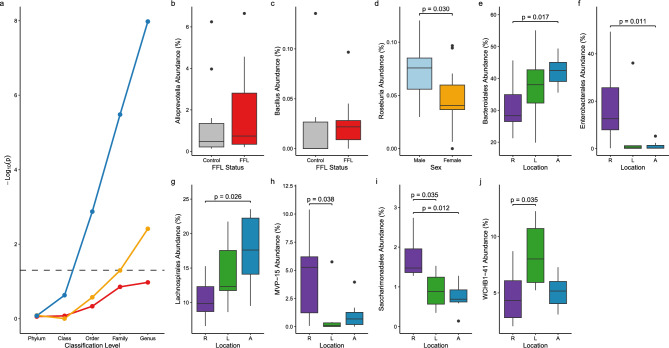
Line plot of taxonomic classification levels (phylum to genus) and -log_10_(p) of the *p*-values from the Kruskal–Wallis tests for FFL status (red), sex (yellow), and geographical location (blue) (**a**). The dashed line represents the *p*-value threshold at 0.05. Boxplots comparing FFL Status at the genera *Alloprevotella* (**b**) and *Bacillus* (**c**). Boxplot comparing Sex at the genus *Roseburia* (**d**). Boxplots of relative abundances for Richmond (R), Langley (L), and Abbotsford (A) for the six taxa Bacteroidales I, Enterobacterales (**f**) Lachnospirales (**g**), MVP-15 (**h**), Saccharimonadales (**i**), and WCHB1-41 (**j**). The boxplots outline the *p*-values of all statistically significant (*p* < 0.05) differences as determined by the Wilcoxon rank sum test.

The overall relative abundance at all taxonomic ranks were compared in FFL and healthy horses using the Kruskal–Wallis test. No statistically significant differences at any of the taxonomic levels (phylum: *p* = 0.877, class: *p* = 0.839, order: *p* = 0.464, family: *p* = 0.140, and genus: *p* = 0.106) between FFL horses and healthy controls indicated in Fig. 4a (red). Given prior findings suggested that the genera *Alloprevotella* and *Bacillus* have differences in FFL status, we further investigated their abundances. The Wilcoxon rank sum test was used to compare the genera and found no differences. Specifically, the median relative abundance of *Alloprevotella* accounted for 0.474 ± 1.130% in control horses and 0.737 ± 2.465% in FFL horses (*p* = 0.202, Fig. 4b), whereas *Bacillus* accounted for 0 ± 0.027% in control horses and 0.023 ± 0.019% in FFL horses (*p* = 0.327, Fig. 4c). The taxonomic lineages up to phylum of both genera were compared using the Wilcoxon rank sum test and found to have no statistical differences (*p* > 0.05).

### Sex microbial composition comparison

The overall relative abundance at all taxonomic levels were compared between male and female horses using the Kruskal–Wallis test. All levels, except for genus (*p* = 0.003) were non-significant (phylum: *p* = 0.817, class: *p* = 0.992, order: *p* = 0.266, and family: *p* = 0.051) as depicted in Fig. 4a (yellow). The Wilcoxon rank sum test was used to compare the relative abundance for each taxon. A statistically significant difference in only the genus *Roseburia* (*p* = 0.030) was found between male (0.04 ± 0.02%) and female (0.08 ± 0.03%) horses (Fig. 4d).

### Geographical location microbial composition comparison

The overall differences in the relative abundance between geographical locations was assessed using the Kruskal–Wallis test. We found no statistical differences at the phylum (*p* = 0.832) and class (*p* = 0.234) taxonomic levels, however, there were statistical differences at the order (*p* = 0.001), family (*p* = 3.31 × 10^−6^), and genus (*p* = 1.05 × 10^−8^) taxonomic levels, as illustrated in Fig. 4a (blue). Further investigation on the individual taxa was done within the order taxonomic level using the Wilcoxon rank sum test. We found statistically significant differences in the relative abundance between Richmond, Langley, and Abbotsford at the orders Bacteroidales (Richmond vs Abbotsford: *p* = 0.017), Enterobacterales (Richmond vs Abbotsford: *p* = 0.011), Lachnospirales (Richmond vs Abbotsford: *p* = 0.026), MVP-15 (Richmond vs Langley: *p* = 0.038), Saccharimonadales (Richmond vs Langley: *p* = 0.035, Richmond vs Abbotsford: *p* = 0.001), and WCHB1-41 (Richmond vs Langley: *p* = 0.035). Boxplots outlining the relative abundance at each of the three locations for each taxon are depicted in Fig. 4e-j. Other comparisons between these three locations for the six taxa were not statistically significant (*p* > 0.05) as outlined in Table 2.

**Table 2:** Results specific orders between the geographical locations Richmond (R), Langley (L), and Abbotsford (A) in which significant differences were indicated in Fig. 4b–g.

Order	Median ± IQR relative abundance			Wilcoxon rank sum test *P*-values		
	Richmond	Langley	Abbotsford	R vs L	R vs A	L vs A
Bacteroidales	28.32 ± 8.45	38.05 ± 10.42	42.51 ± 5.98	0.365	0.017	0.455
Enterobacterales	12.62 ± 17.74	0.61 ± 1.10	0.22 ± 1.19	0.101	0.011	0.945
Lachnospirales	9.86 ± 3.61	12.31 ± 5.82	17.61 ± 8.14	0.234	0.026	0.234
MVP-15	5.27 ± 4.97	0.09 ± 0.33	0.69 ± 1.08	0.038	0.097	0.350
Saccharimonadales	1.48 ± 0.60	0.89 ± 0.68	0.69 ± 0.30	0.035	0.001	0.628
WCHB1-41	4.33 ± 3.28	8.01 ± 4.79	5.16 ± 1.97	0.035	0.710	0.051

## Discussion

To test the hypothesis that dysbiosis is associated with FFL, recent studies have compared the fecal microbial composition of horses with FFL against healthy animals. However, the results of these studies are conflicting. While Lausten et al.^1^ and Schoster et al.^3^ conclude no statistical significance in microbial composition between healthy horses and those with FFL, Lindroth et al.^5^ found statistical differences in the genera *Alloprevotella* and *Bacillus*. These three studies examining the association between FFL and the microbiome have exclusively been conducted in Europe. To our knowledge, our current study is the first to investigate the correlation between dysbiosis and FFL in Canadian horses. Our results support the conclusion that there is no significant difference between the microbiome of feces from FFL animals and controls. These findings support that dysbiosis is likely not associated with FFL. Our results show that fecal samples from FFL and control animals have similar microbial community composition and structure when visualized using PCoA. Statistical analysis using ANOSIM and PERMANOVA substantiate these results. Furthermore, no individual taxa were identified as having any significant differences in relative abundance using Wilcoxon rank sum hypothesis testing. These results strongly suggest no difference between the microbial composition of the FFL and healthy horses in this Canadian study. Our findings are consistent with Laustsen et al.^1^ who conducted a similar study while monitoring symptom severity after fecal transplants and Schoster et al.^3^ who compared FFL and healthy horses in both the autumn and spring seasons.

The current study did however identify differences in microbial composition when comparing sex and some of the geographical locations. Differences between male and female horses was only visible when comparing individual taxa at the genus level and not in alpha and beta diversity metrics. Specifically, the genus *Roseburia* was found to be more abundant in female horses. Genus *Roseburia* is known to be a butyrate-producing, gram-positive anaerobic bacteria^39^. Specifically, the species *R. intestinalis* has been labelled as a beneficial gut organism and is considered to have therapeutic role in various human diseases^39^. Differences in microbial communities between sex of horses have been previously reported, however, indicating different taxa^40^. Differences between geographical location were also evident from the ANOSIM and PERMANOVA analyses identifying differences between the Richmond and Abbotsford locations. The PCoA plots indicated that a relatively low percentage of variation among groups could be accounted for by geographic location, despite the apparent clustering of Richmond and Abbotsford groups. This was further confirmed when comparing taxa at each taxonomic rank, where we see differences in microbial composition at the order classification level and below. Specifically, the orders Bacteroidales, Enterobacterales, Lachnospirales, and Saccharimonadales had different relative abundances between Richmond and Abbotsford horses. We also visualize differences in the orders MVP-15, Saccharimonadales, and WCHB1-41 between Richmond and Langley. Richmond and Abbotsford are the farthest from each other within the study group, being 66 km apart and Richmond and Langley are the second farthest distance at 41 km apart from each other. Microbial community differences have also been reported in different geographical locations, however, at a much greater magnitude than the distances between the Richmond, Langley, and Abbotsford locations^41^.

Limitations of this study could provide sources of error that prevent the detection of small, yet significant, differences in the microbiota of horses with FFL. For instance, variance introduced from collecting samples from different geographical locations and the relatively small sample size. The microbial population differences found between the locations could be masking minor changes between horses with FFL and healthy controls. However, in comparison to other studies between gut microbiome and FFL in horses^1,3,5^, the locations that the samples analyzed in the present study were taken from a smaller land area. While the sample size is also relatively small, it is difficult to obtain more samples due to prevalence of the condition. FFL severity between cases may have also obscured the detection of differences as samples collected from horses with both acute and chronic FFL symptoms were grouped together. It should also be noted that Lindroth et al.^5^ found differences using a much larger sample size in comparison to this study and others that these results are consistent with^1,3^. Additionally, diet is known to be a factor that contributes to variations in microbial composition^42^. As horses were from different farms, their diets varied.

FFL could be a condition that is associated with multiple factors^3^. Some reports point towards stress^4^ or disturbances associated by gastric disorders^43^ or feeding practices^42^, in addition to microbial composition^3,5^. To assess these factors, it may be valuable to do a longitudinal study monitoring diet, stress, and health, collecting fecal matter over a longer period to observe changes in microbial composition over the course of the experiment. Microbial composition and diversity may not be the central factor that affects FFL, but rather simply a contributor towards the metabolomic environment that both the horse and microbiota generate within the hindgut. Recently, in human microbiome research, there has been a shift in focus from microbial composition and diversity to functional aspects of the microbiota^44^. In concordance with this, fecal transplants from clinically healthy donors containing microbes and metabolites were shown to reduce the severity of FFL symptoms in horses^1^. Transcriptomic analyses of both the horse and its microbiota might illuminate possible tissue-microbe interactions that lead to FFL. Recent studies have determined transcriptomic profiles in equine gastrointestinal tissues; however, no studies have involved transcriptomics of the equine microbiota^45,46^.

The results of this study indicated no differences in the microbial composition and diversity between healthy and FFL horses, consistent with other findings^1,3^. However, differences in the composition and diversity of samples between sex and the geographic locations Richmond and Abbotsford as well as Richmond and Langley were found. It is important to find the root cause of FFL, so that effective treatments can also be determined. Further research is needed to determine whether dysbiosis or other factors lead to FFL, and exactly what role the gut microbiota and its metabolites play.

### Supplementary Information

Supplementary Legends.

Supplementary Figure S1.

Supplementary Figure S2.

Supplementary Figure S3.

Supplementary Figure S4.

Supplementary Table S1.

## Data Availability

The sequencing data that supports the findings of this study are readily accessible on NCBI’s Sequence Read Archive repository at https://www.ncbi.nlm.nih.gov/sra/PRJNA1034666, BioProject accession number PRJNA1034666.
