# Supplementary material for: Dysbiosis not observed in Canadian horses with free fecal liquid (FFL) using 16S rRNA sequencing
Source: Sci Rep. 2024 Jun 5;14:12903. doi: 10.1038/s41598-024-63868-1 (PMC11153561; doi:10.1038/s41598-024-63868-1)
Supplement: Supplementary file 1 — Supplementary Legends. [file 41598_2024_63868_MOESM1_ESM.docx]

**Figure Legends**

**Supplementary Figure S1.** Bar charts illustrating the mean relative abundance of families (a and b) and genera (c and d). between FFL status (a and c) and geographical location (b and d). All named taxa displayed have a mean relative abundance above 1%.

**Supplementary Figure S2.** Bar charts outlining the relative abundance of phyla (a), classes (b), orders (c), families (d), and genera (e) for all 25 horses in the study. The horses in these bar charts are grouped by FFL status. All named taxa displayed have a mean relative abundance above 1%.

**Supplementary Figure S3.** Bar charts illustrating the relative abundance of phyla (a), classes (b), orders (c), families (d), and genera I between male and female horses. All named taxa displayed have a mean relative abundance above 1%.

**Supplementary Figure S4.** Principal coordinate analyses (PCoA) of the microbial composition and diversity between sex using the Bray-Curtis index (a) and the Jaccard index (b). Males are represented as the light blue colour and females are represented by the light orange colour.
