# Supplementary figures and images for: Dysbiosis not observed in Canadian horses with free fecal liquid (FFL) using 16S rRNA sequencing
Source: Sci Rep. 2024 Jun 5;14:12903. doi: 10.1038/s41598-024-63868-1 (PMC11153561; doi:10.1038/s41598-024-63868-1)

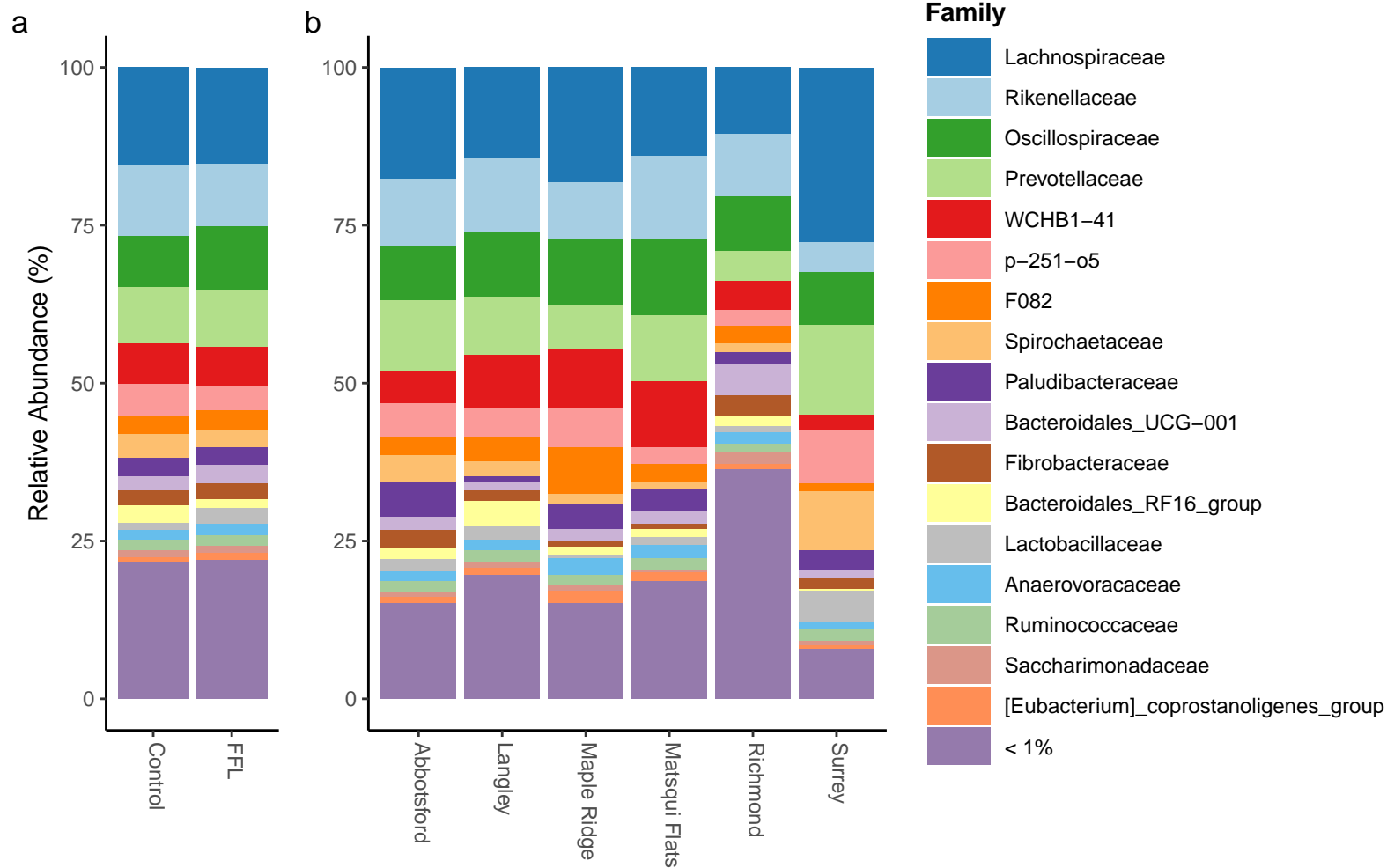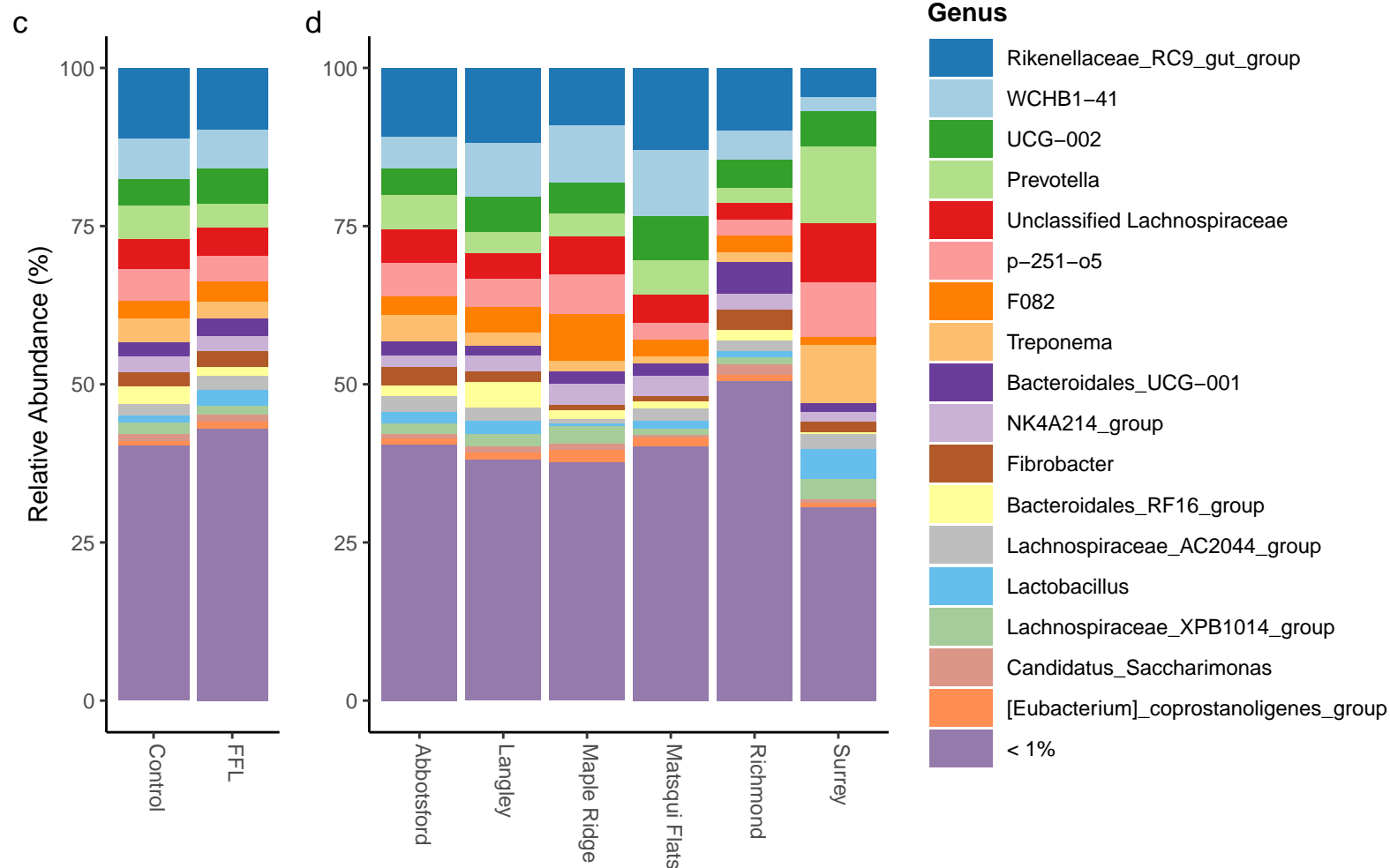

Supplement: Supplementary file 2 — Supplementary Figure S1. [file 41598_2024_63868_MOESM2_ESM.pdf]

a

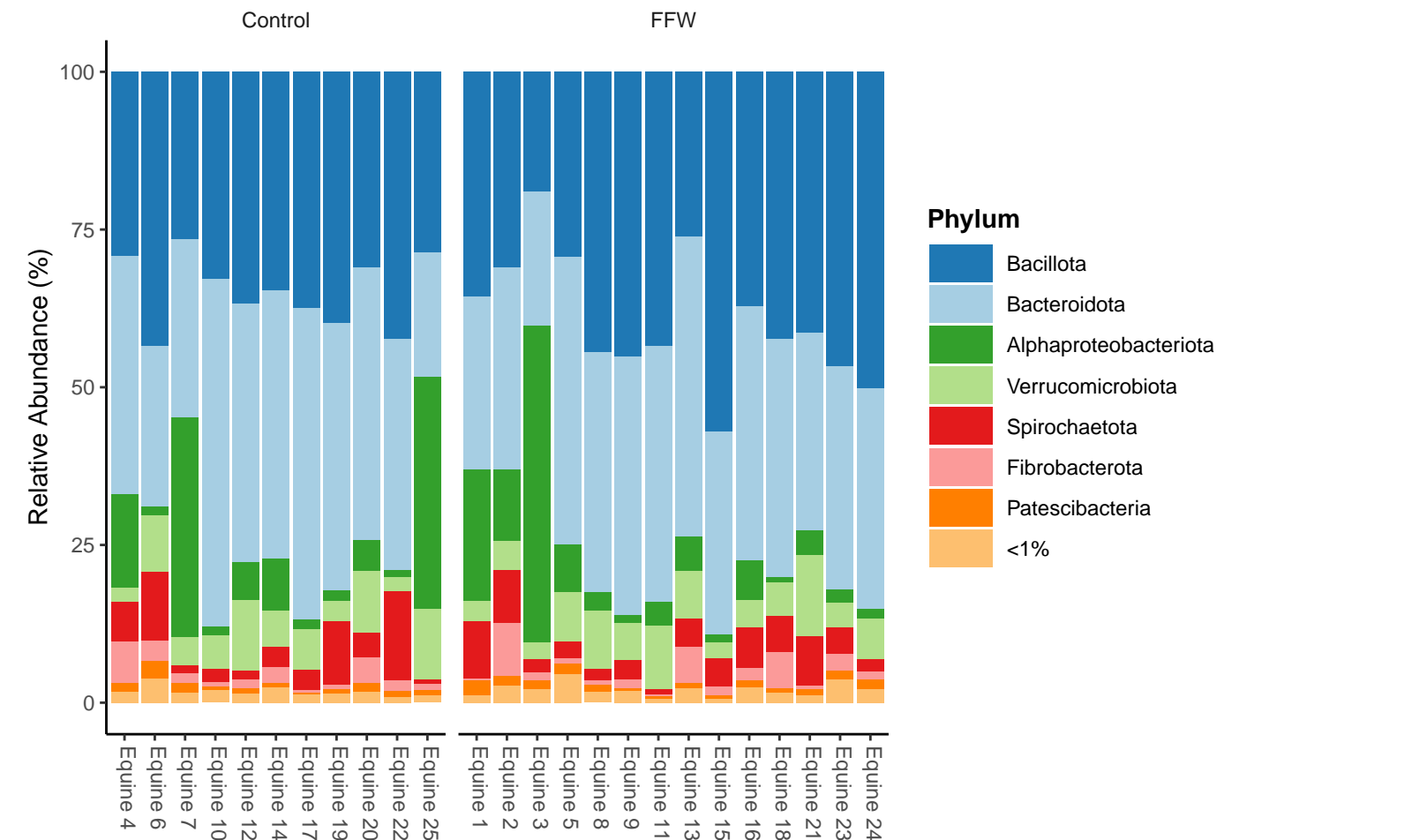

b

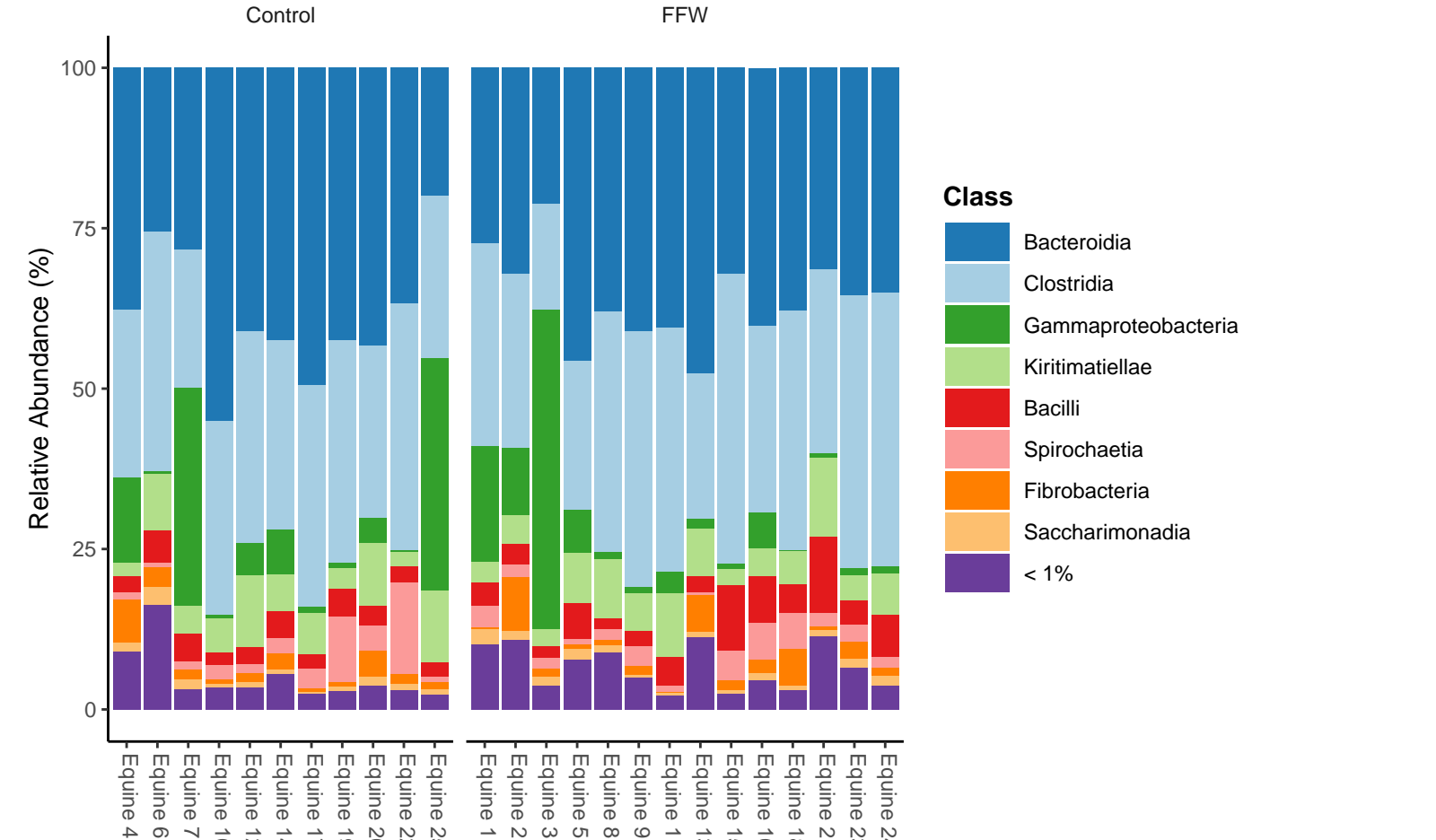

c

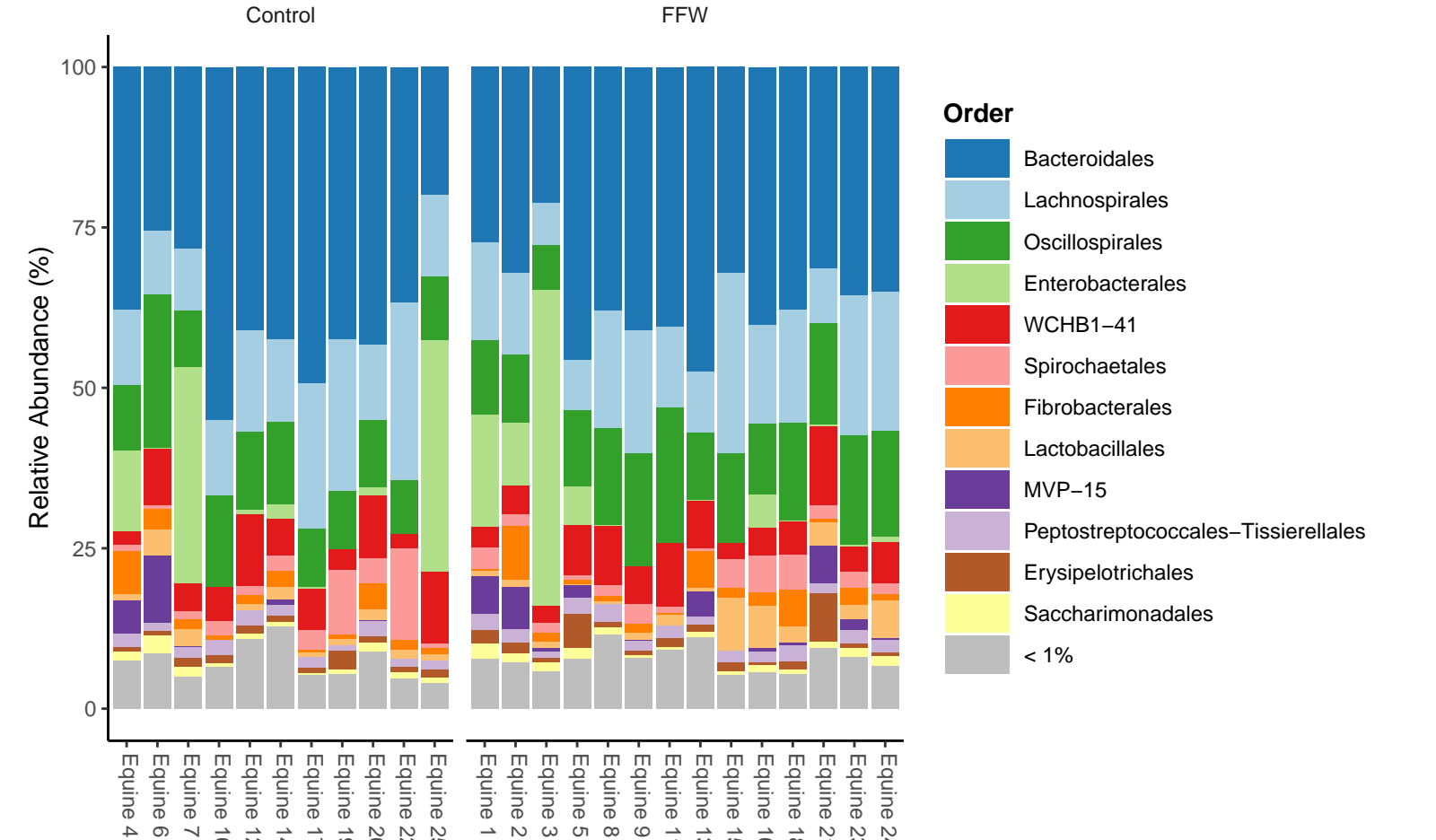

d

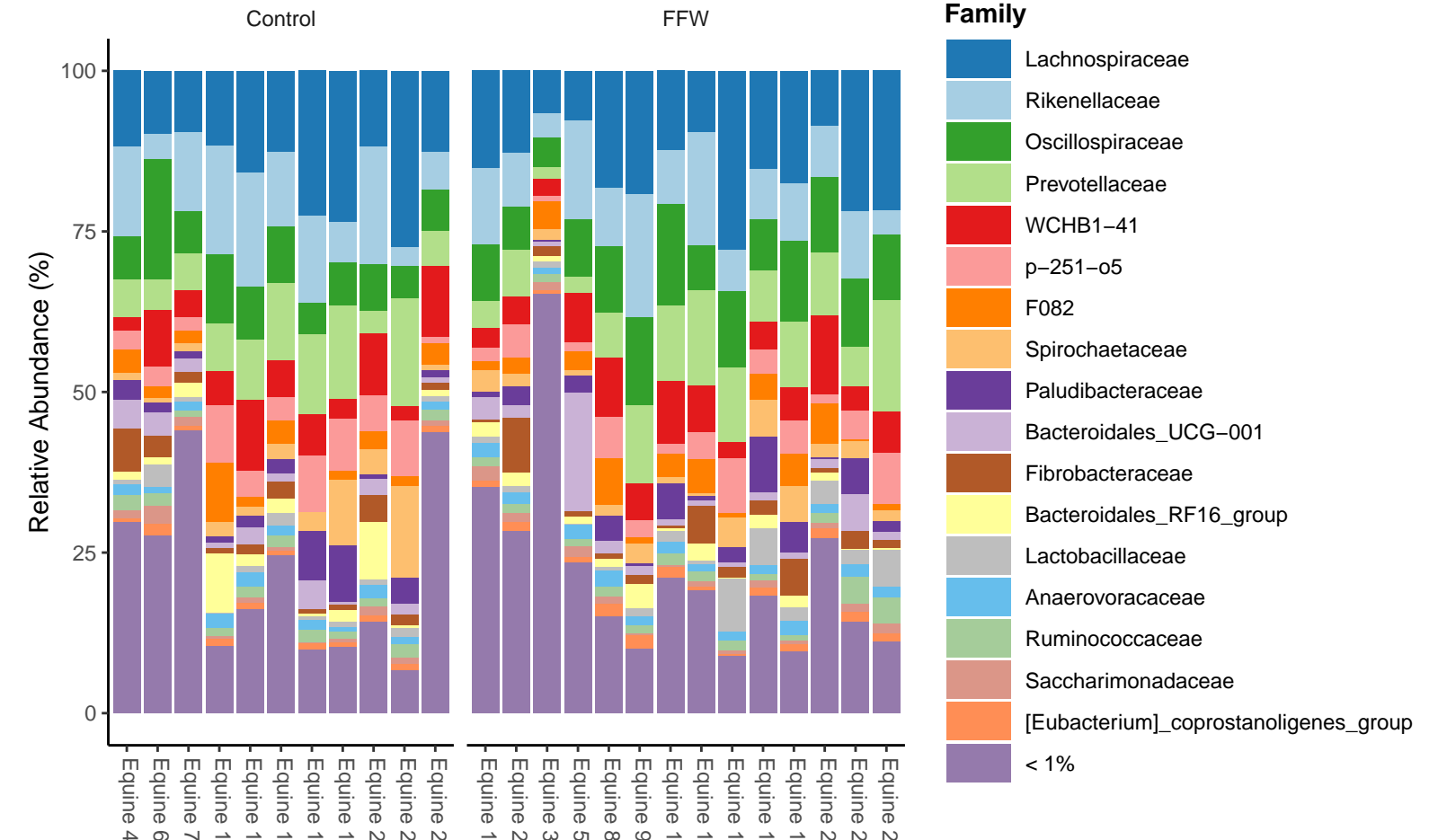

e

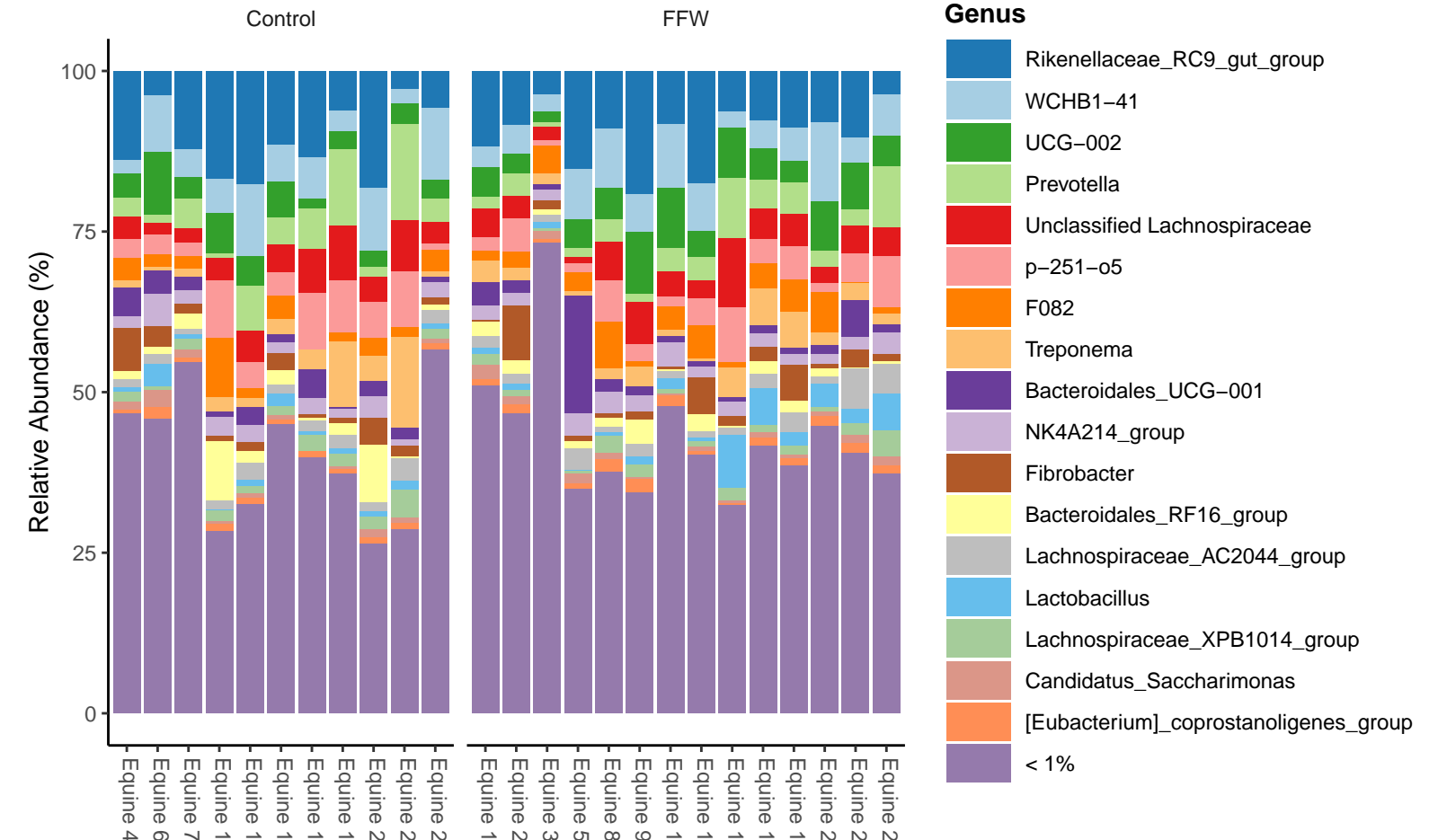

Supplement: Supplementary file 3 — Supplementary Figure S2. [file 41598_2024_63868_MOESM3_ESM.pdf]

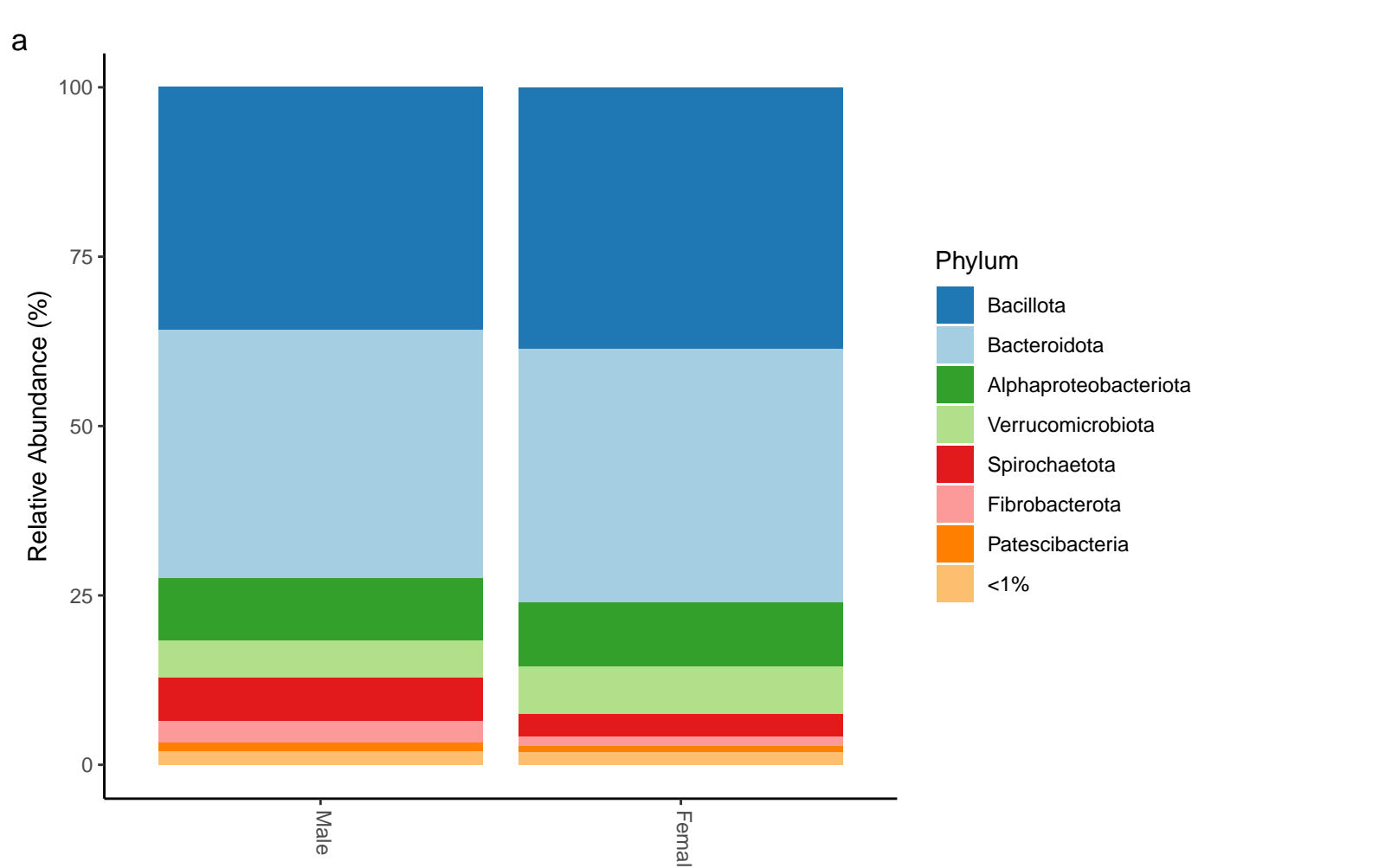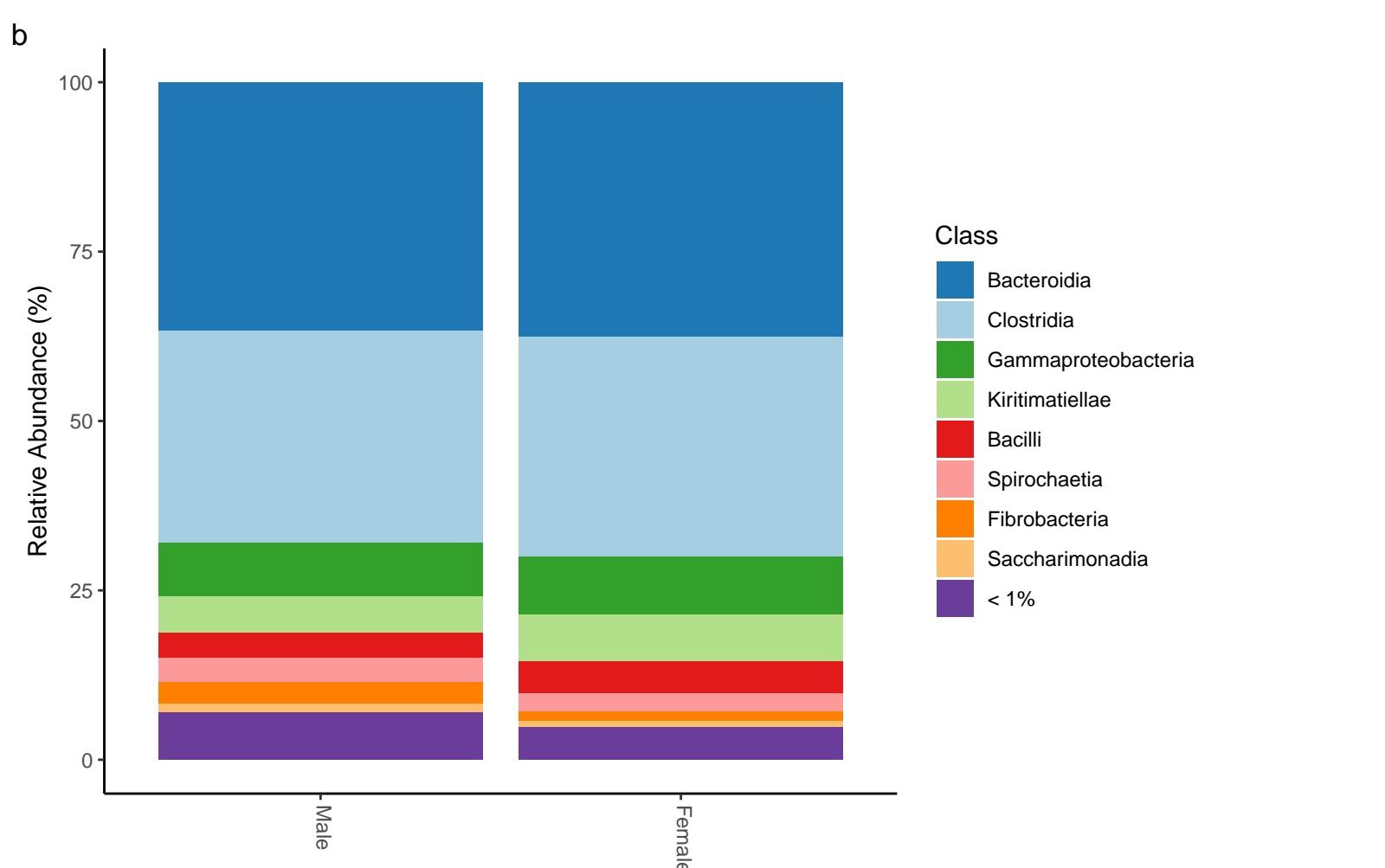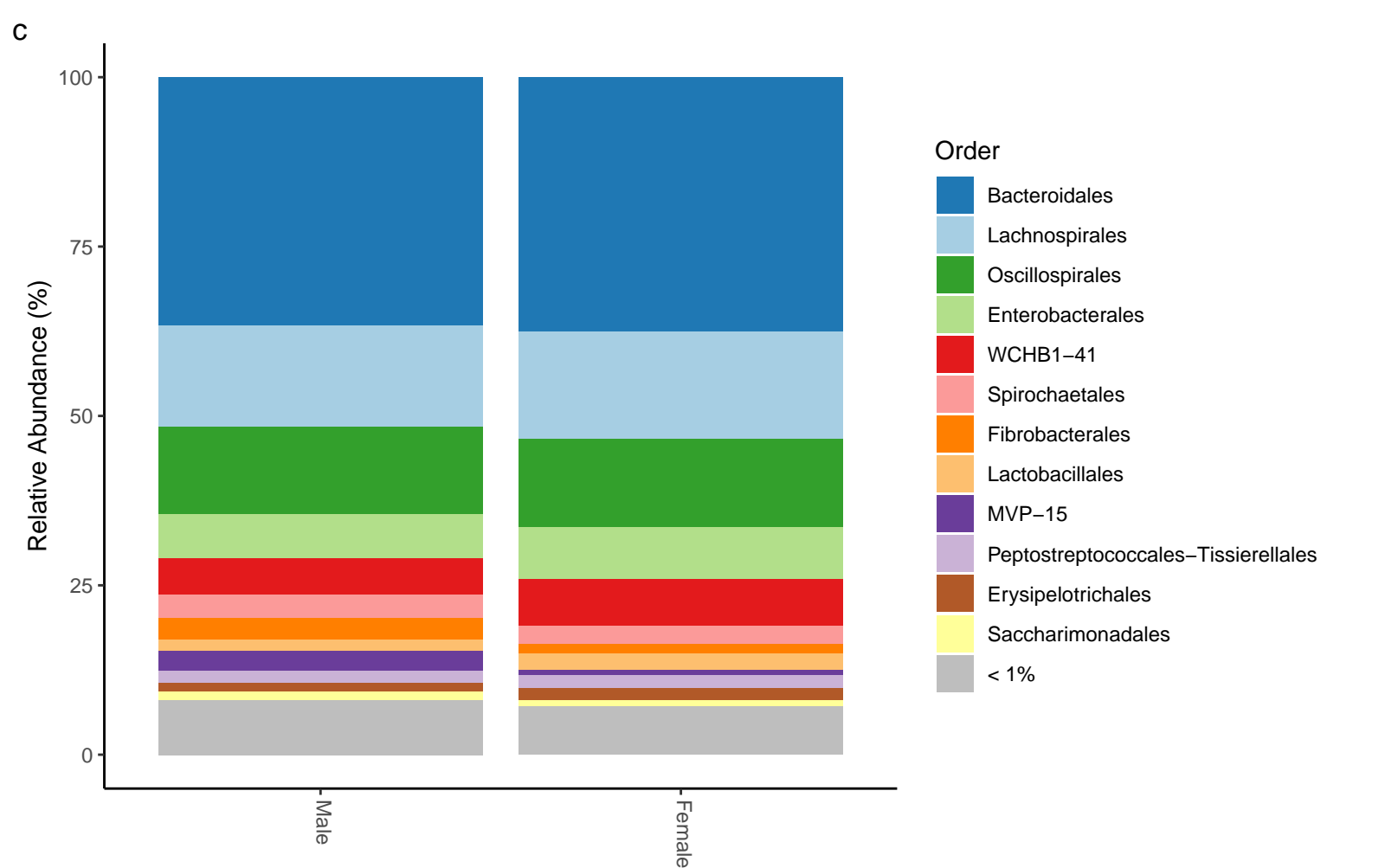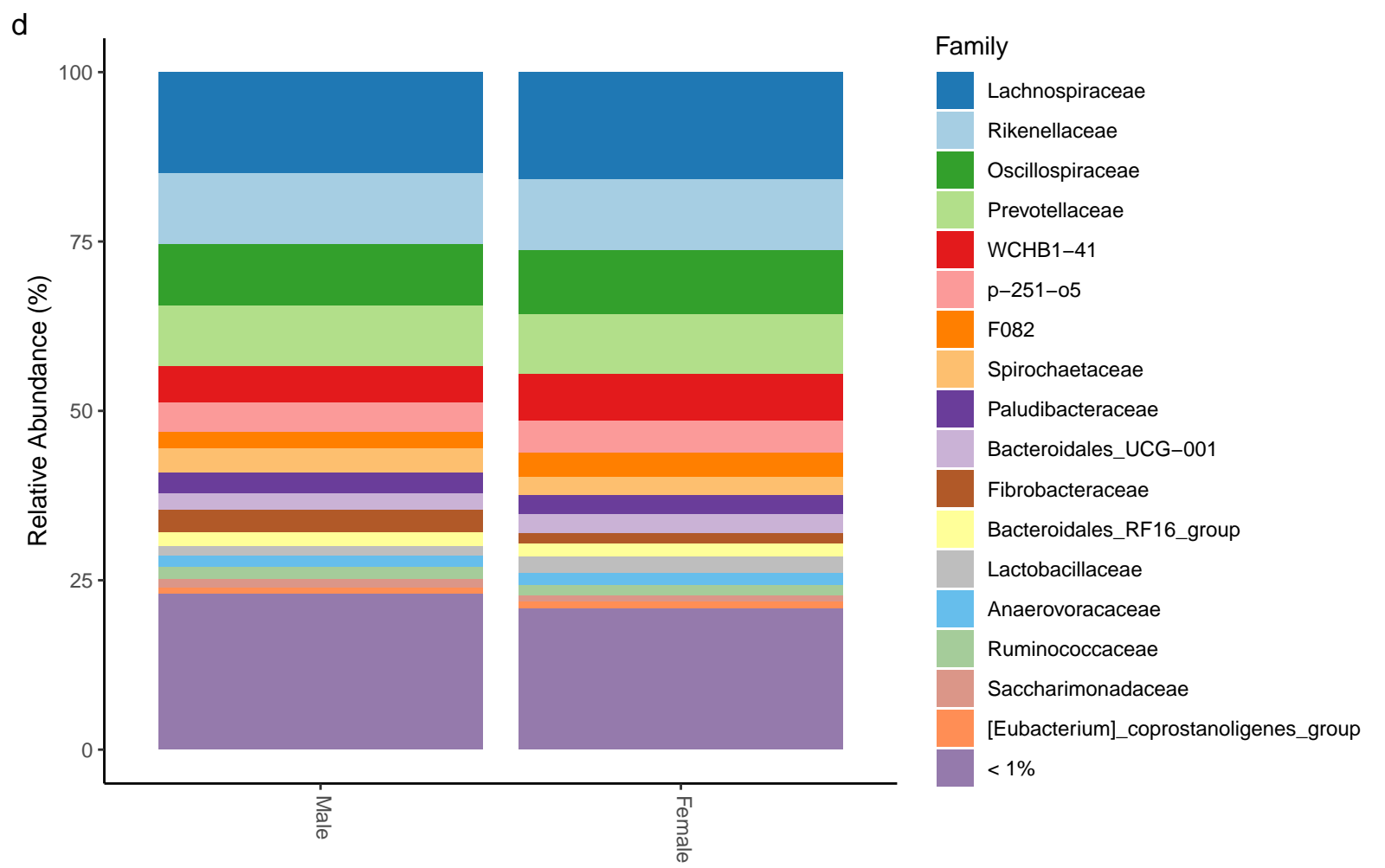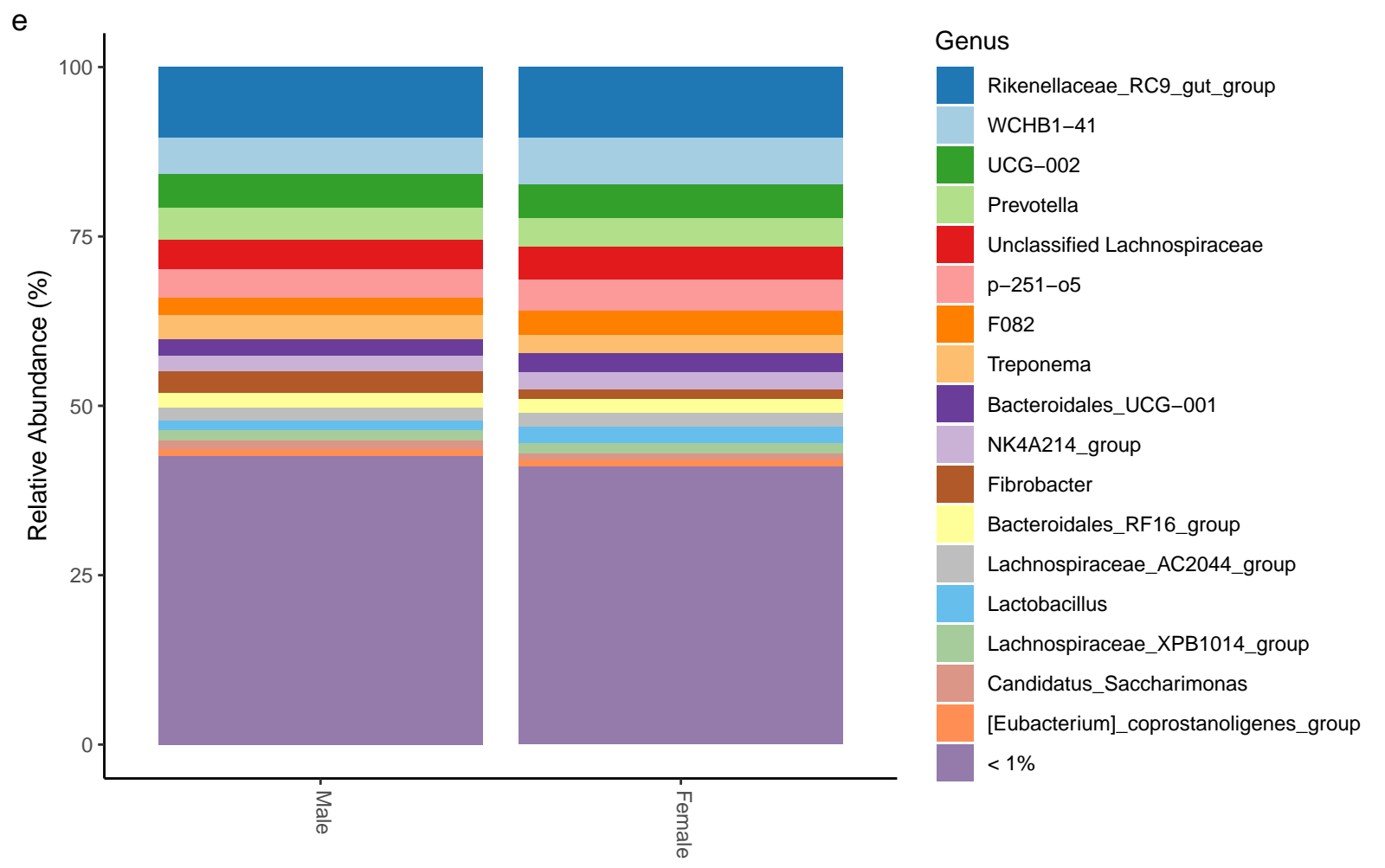

Supplement: Supplementary file 4 — Supplementary Figure S3. [file 41598_2024_63868_MOESM4_ESM.pdf]

a

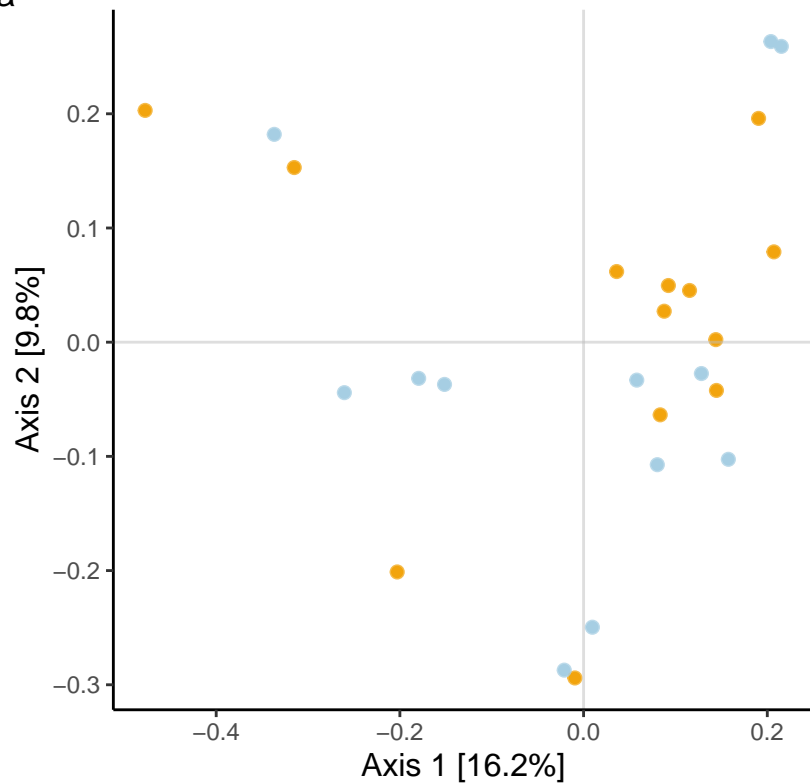

b

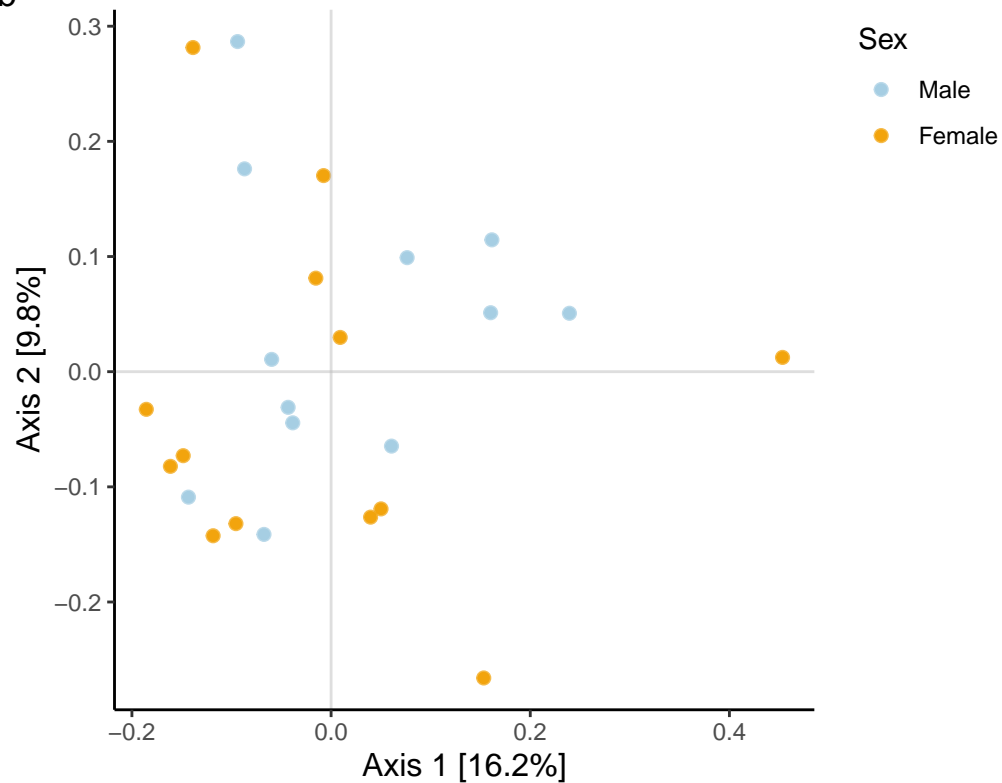

Supplement: Supplementary file 5 — Supplementary Figure S4. [file 41598_2024_63868_MOESM5_ESM.pdf]
